# Supplementary material for: The causal role of circulating inflammatory markers in osteoporosis: a bidirectional Mendelian randomized study
Source: Front Immunol. 2024 Jul 18;15:1412298. doi: 10.3389/fimmu.2024.1412298 (PMC11291241; doi:10.3389/fimmu.2024.1412298)
Supplement: Supplementary file 1 [file DataSheet_1.zip › supplementary material/CIM-OSTEOPOROSIS /GCST90274800.csv_forest.pdf]

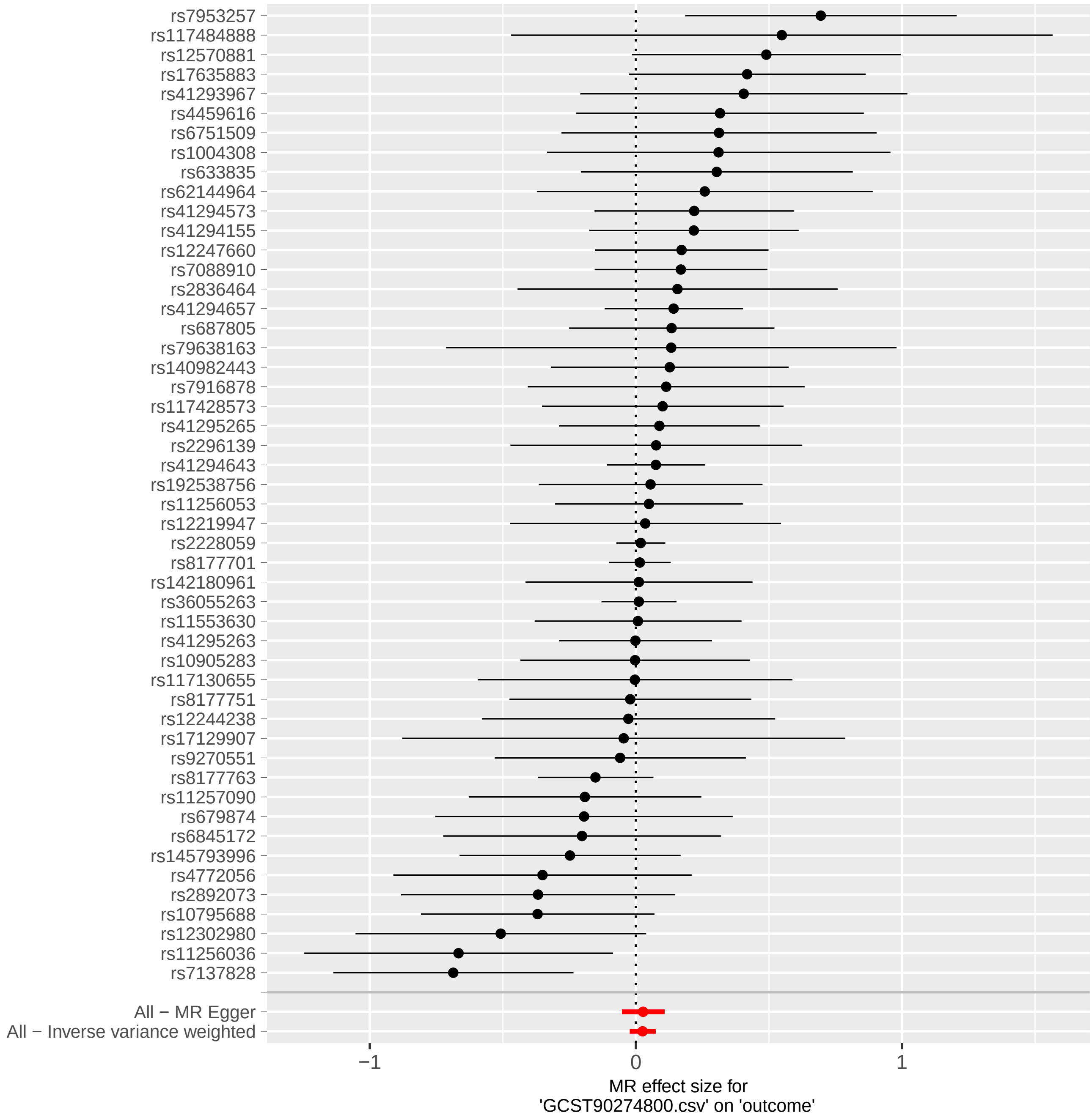
rs7953257
rs117484888
rs12570881
rs17635883
rs41293967
rs4459616
rs6751509
rs1004308
rs633835
rs62144964
rs41294573
rs41294155
rs12247660
rs7088910
rs2836464
rs41294657
rs687805
rs79638163
rs140982443
rs7916878
rs117428573
rs41295265
rs2296139
rs41294643
rs192538756
rs11256053
rs12219947
rs2228059
rs8177701
rs142180961
rs36055263
rs11553630
rs41295263
rs10905283
rs117130655
rs8177751
rs12244238
rs17129907
rs9270551
rs8177763
rs11257090
rs679874
rs6845172
rs145793996
rs4772056
rs2892073
rs10795688
rs12302980
rs11256036
rs7137828
All − MR Egger
All − Inverse variance weighted
−1
0
1
MR effect size for
'GCST90274800.csv' on 'outcome'
